# TVA-based assessment of visual attentional functions in developmental dyslexia

**DOI:** 10.3389/fpsyg.2014.01172

**Published:** 2014-10-16

**Authors:** Johanna Bogon, Kathrin Finke, Prisca Stenneken

**Affiliations:** ^1^Department of Experimental Psychology, University of RegensburgRegensburg, Germany; ^2^Department of Psychology, General and Experimental Psychology/Neuro-Cognitive Psychology, Ludwig-Maximilians-Universität MünchenMunich, Germany; ^3^Department of Speech and Language Pathology, University of CologneCologne, Germany

**Keywords:** developmental dyslexia, visual attention, processing speed, visual short term memory, spatial bias, top–down control, whole- and partial-report task

## Abstract

There is an ongoing debate whether an impairment of visual attentional functions constitutes an additional or even an isolated deficit of developmental dyslexia (DD). Especially performance in tasks that require the processing of multiple visual elements in parallel has been reported to be impaired in DD. We review studies that used parameter-based assessment for identifying and quantifying impaired aspect(s) of visual attention that underlie this multi-element processing deficit in DD. These studies used the mathematical framework provided by the “theory of visual attention” (Bundesen, 1990) to derive quantitative measures of general attentional resources and attentional weighting aspects on the basis of behavioral performance in whole- and partial-report tasks. Based on parameter estimates in children and adults with DD, the reviewed studies support a slowed perceptual processing speed as an underlying primary deficit in DD. Moreover, a reduction in visual short term memory storage capacity seems to present a modulating component, contributing to difficulties in written language processing. Furthermore, comparing the spatial distributions of attentional weights in children and adults suggests that having limited reading and writing skills might impair the development of a slight leftward bias, that is typical for unimpaired adult readers.

## VISUAL ATTENTION AND DEVELOPMENTAL DYSLEXIA (DD)

Visual attentional functions are currently discussed as being related to developmental dyslexia (DD), a disorder in written language acquisition, that cannot be explained by age, visual sensory problems, or inadequate reading instruction (World Health Organization, 2011). Due to increasing evidence for an underachievement of people with DD in many attention-based tasks, it is debated whether an impairment of visual attentional functions constitutes an additional or even an isolated deficit of subgroups of DD (Heim et al., 2008; Menghini et al., 2010).

Especially performance in tasks that require the processing of multiple visual elements in parallel seems to be impaired in DD (Valdois et al., 2003, 2012; Pammer et al., 2004; Hawelka and Wimmer, 2005; Bosse et al., 2007; Jones et al., 2008; De Luca et al., 2010; Romani et al., 2011; Lobier et al., 2012). This multi-element processing deficit was mainly assessed using tasks based on Averbach and Sperling (1968) where participants have to report as many letters as possible (whole-report) or only precued ones (partial-report) from briefly displayed visual arrays. For example, Bosse et al. (2007) revealed that multi-element processing was significantly impaired in children with DD and that this deficit accounted for a substantial variance in reading speed and accuracy. Furthermore, reduced multi-element processing in participants with DD was associated with an increased number of rightward fixations in text reading and of eye movements in word and pseudoword reading (Hawelka and Wimmer, 2005; Prado et al., 2007). Importantly, multi-element processing performance depends on distinct attentional functions. Poor performance could stem from deficient general attentional resources, involving visual processing speed, and/or visual short-term memory (VSTM). Furthermore, it could be related to selectivity changes, involving spatial distribution of attentional weights and/or top–down control.

One influential theoretical concept focusses on general resource limitations. It is suggested that people with DD have a reduced visual attentional span, assessed as the amount of elements that can be reported from a briefly displayed array (Bosse et al., 2007; Prado et al., 2007; Valdois et al., 2011, 2012; Lobier et al., 2012). Critically, this deficit could be caused by an enhanced visual threshold, a reduction in visual processing speed or VSTM storage capacity, or by a combination of such deficits. A delayed start or a slower rate of encoding the elements of a briefly displayed multi-element array should both lead to a reduced number of elements that enter VSTM before the display disappears. Otherwise, a limitation of the maximum number of elements that can be stored in VSTM, could also account for a reduced visual attentional span.

Additionally or alternatively, impaired attentional selectivity aspects, i.e., changes in spatial attention and/or inefficiency in filtering information could also account for deficient multi-element processing. Indeed, people with DD might not show a normal slight leftward attentional bias. It is known that patients with hemi-neglect following right parietal damage show a rightward attentional bias, which is shown, for example, in rightward deviations in line bisection tasks. Normal participants instead deviate slightly, but reliably toward the left when bisecting lines (Bowers and Heilman, 1980) and also show leftward bias in speeded lateralized stimulus detection. This behavior is termed “pseudoneglect.” A number of studies indicate that participants with DD do not show pseudoneglect (Facoetti et al., 2000, 2003; Facoetti and Molteni, 2001; Hari et al., 2001; Buchholz and Davies, 2005; Sireteanu et al., 2005; Liddle et al., 2009; Ruffino et al., 2010; Waldie and Hausmann, 2010; Ziegler et al., 2010; Stenneken et al., 2011). Reduced efficiency of top–down controlled selection could also account for poor multi-element processing. People with DD might be especially prone to interference (Sperling et al., 2005; Roach and Hogben, 2007; Moores et al., 2011; Stevens et al., 2013) and such susceptibility to distracting information might reduce the amount of relevant elements encoded from briefly displayed multi-element arrays.

In this review, we explore the role of visual attention functions for impaired multi-element processing in DD. A critical methodological challenge is to identify and quantify the impaired function(s). Recently, a number of studies have used a parameter-based account of visual attention assessment (Dubois et al., 2010; Stenneken et al., 2011; Lobier et al., 2013; Bogon et al., 2014). In these studies, the formal framework provided by the “theory of visual attention” (TVA; Bundesen, 1990, 1998) was used to derive quantitative estimates of the individual capabilities of a participant in selecting visual information. This was done by computational modeling of behavioral performance in two simple, psychophysical tasks, i.e., whole- and partial-report of briefly presented letter arrays.

## IDENTIFICATION AND QUANTIFICATION OF DISTINCT ATTENTIONAL PARAMETERS BY MEANS OF THE TVA

Within the TVA framework, the efficiency of visual selection performance of a given participant can be described on the basis of a set of mathematically independent, quantitative measures of attentional components (for a comprehensive description of TVA see Bundesen, 1990; for a formal description and TVA equations see Kyllingsbæk, 2006). TVA assumes that objects from a briefly presented array are processed in parallel and compete for selection into a VSTM store. Only objects can be reported that reach the store before its storage capacity is exhausted and before iconic memory of the array vanishes. The resulting race among objects can be biased in such a way that some objects are favored for selection, based either on stimulus-driven, “bottom–up” or on intentional, “top–down” factors. The probability of selection is determined (i) by the participant’s individual minimal effective exposure duration, the visual perception threshold *t0*, (ii) by an object’s processing rate, which depends on the relative attentional weight it receives, and (iii) by the capacity of the VSTM store *K* (if the store is filled, selection terminates). TVA provides parameters for characterizing the general processing efficiency of the information processing system (minimal effective visual exposure duration, processing speed, and VSTM storage capacity), and for characterizing attentional selectivity (top–down control and spatial distribution of attention).

**FIGURE 1:**
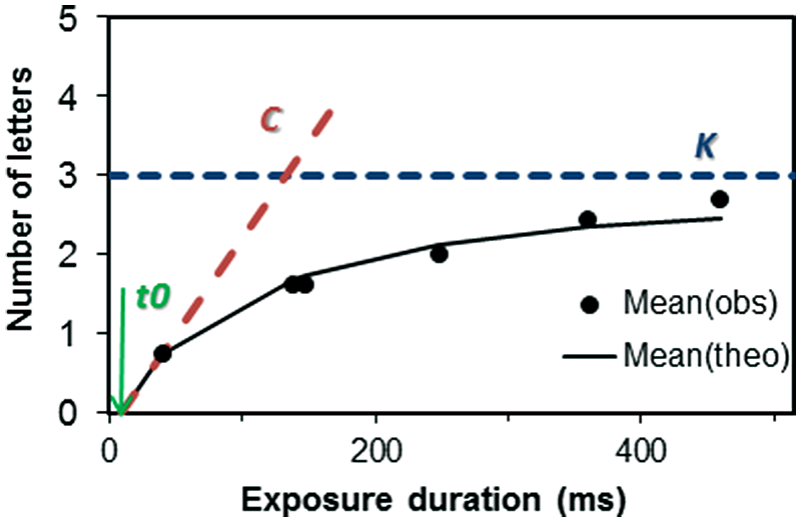
**Whole-report performance for a typical adult participant (*K* = 3.0 elements; *C* = 23.0 elements/s; *t0* = 9 ms)**. The mean number of correctly reported letters is shown as a function of exposure duration. The solid line represents the best fit from the “theory of visual attention” (TVA) to the observations. The minimum effective exposure duration *t0* denotes the visual perception threshold. The estimate of the visual VSTM storage capacity *K* is marked by the dashed horizontal line, and that of the visual perceptual processing speed *C* by the skewed dashed line. *Mean(obs)* = observed number of letters reported correctly; *Mean(theo)* = theoretically predicted numbers of letters reported correctly; *t0* = minimal effective exposure duration (in ms), *K* = VSTM storage capacity (in number of letters), *C* = perceptual processing speed (in number of letters/s).

In TVA-based assessment, the general information processing efficiency is assessed within a whole-report task, in which subjects are briefly presented with multiple stimuli and have to identify as many as possible. The probability of identification is modeled by an exponential growth function (see **Figure 1**), in which the visual perception threshold (parameter *t0*: minimal effective exposure duration in ms), the growth parameter reflects the rate at which the stimuli (objects) can be processed (parameter processing speed *C*: number of element/s), and the asymptote of the growth function indicates the maximum number of objects that can be represented within VSTM (parameter VSTM storage capacity *K*). Thus, estimating these parameters of interest here permits to further differentiate if a deficient visual span performance (e.g., Bosse et al., 2007) in DD is caused by deficiencies in visual perception threshold or visual encoding speed, by storage capacity problems or a combination of these factors.

Furthermore, TVA-based assessment allows to individually estimate critical selectivity aspects of attention of interest here: spatial laterality (parameter spatial distribution of attention *w*_λ_) and efficiency in prioritizing targets over distractors (parameter top–down control α). Parameter *w*_λ_ is derived in report tasks that involve trials with presentation of stimuli in only one and trials with stimuli in both hemifields. Based on report individual accuracy differences in bilateral vs. unilateral displays, the TVA model produces estimates of attentional weights *w_i_* separately for the left (*w*_L_) and the right hemifield (*w*_R_), and *w*_λ_ is then computed as *w*_L_/(*w*_L_ + *w*_R_). Hence, a value of *w*_λ_ = 0.5 indicates balanced weighting; values of *w*_λ_ > 0.5 indicate a leftward and values of *w*_λ_ < 0.5 a rightward bias. A slight normal “pseudo-neglect” is indicated by a value of *w*_λ_ > 0.5, because weights for objects to the left of fixation are slightly higher than those for objects to the right. If participants with DD indeed show a reduced or absent pseudoneglect, this would be indicated by significantly higher *w_λ_* values compared to control participants. Parameter α, representing efficiency of top–down attentional control, is estimated from the performance in partial-report tasks, where participants have to report target objects, only, which are prespecified (e.g., with respect to color), whilst ignoring distractors. The parameter indicates the relative attentional weights of distracters compared to targets (*w*_D_/*w*_T_). Impaired top–down control in participants with DD would be indicated by higher α-values compared to control participants, indicating relatively more attentional weight allocated to distracters. In sum, estimating these selectivity parameters permits the exploration of the potential contribution of lateralized deficits or inefficiency of top–down control to impairments found in DD.

## TVA-BASED STUDIES ON DD

The first TVA-based study that aimed to disentangle the attention deficits underlying impaired multi-element processing was a case study on two children with DD (Dubois et al., 2010). These children with impaired performance in standardized whole- and partial-report tasks (Valdois et al., 2003, 2004) demonstrated reading and writing difficulties, characterized by a high number of reading and spelling errors and a strikingly reduced reading speed whereas their intellectual abilities were in the normal range. The parameters minimal effective exposure duration *t0*, visual processing speed *C*, VSTM storage capacity *K* and laterality of attentional weighting *w*_λ_ were estimated by modeling whole-report accuracy for both cases and compared to an age-matched group of nine children with typical reading and writing abilities. This revealed a reduction in visual processing speed for both children with DD and, additionally, a reduced VSTM storage capacity for one of them. The two cases did not differ from controls in minimal effective exposure duration and the spatial distribution of attention.

Further insights into the potential contribution of these attentional parameters to dyslexic impairments at a later developmental stage has been provided by a group study on adults with DD. Stenneken et al. (2011) compared TVA parameter estimates in high-achieving young adults (mostly university students) with persisting DD to an age- and education-matched control group. With regard to general attentional resources a profound impairment of visual processing speed *C* was found in the group of adults with DD compared to controls. Moreover, with regard to selectivity aspects, as assessed by a partial-report task, the distribution of spatial attentional weights was found to be different than in controls. The group of normal readers showed the typical, slight leftward bias in spatial attentional weighting (i.e., pseudoneglect, Jewell and McCourt, 2000), that has been documented in unimpaired participants (for TVA-based studies, cf. Bublak et al., 2005; Finke et al., 2005; Habekost and Rostrup, 2006; Matthias et al., 2009, 2010). In contrast, participants with DD did not show this effect; interestingly, the more the spatial lateralization in these participants deviated from that of controls the more severe was their dyslexia, as assessed by a standardized spelling test.

In order to further specify developmental aspects of DD that reconcile aspects of the previous single case study on children and the group study in adults, Bogon et al. (2014) conducted a TVA-based assessment in a group of children with DD and a group of typically developing children matched according to age, educational level, gender, and general intellectual ability. Group-wise comparisons revealed the general attentional processing resource parameters, visual processing speed and VSTM storage capacity, to be impaired in children with DD compared to controls. Moreover, in the group of children with DD, low VSTM storage capacity was significantly related to impaired reading performance. In contrast, the selectivity aspects of visual attention, spatial distribution of attentional weights, and top–down control, were comparable to those of controls.

## DISCUSSION

**FIGURE 2:**
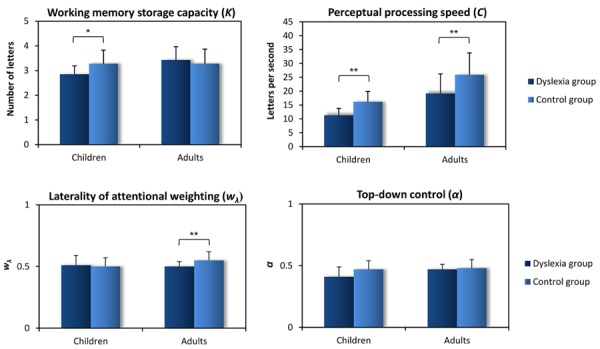
**Average estimates (and SD of the parameter estimates between participants per group) for the TVA parameters in children and adults with developmental dyslexia (DD) and the respective control groups (based on data of Bogon et al., 2014 and Stenneken et al., 2011).** *p < 0.05, **p < 0.01.

Taken together, all TVA-based studies on DD implicate that a reduced perceptual processing speed is the most profound impairment at the core of DD (Dubois et al., 2010; Stenneken et al., 2011; Bogon et al., 2014). The parameter estimates assessed in the group studies are given in **Figure 2**. In both children with DD examined by Dubois et al. (2010), visual processing speed was severely reduced compared to controls while visual threshold *t0* was normal (a somewhat different paradigm was used, resulting in higher absolute values in *C* and *K* compared to **Figure 2**, with similar difference to controls). The speed reduction was replicated at group level, with similar degree in children and adults with DD (Stenneken et al., 2011; Bogon et al., 2014). Again, these studies did not report changes in visual threshold. These findings indicate that when the rate of visual information uptake is abnormally slow, this can hinder the acquisition of normal reading skills. However, the central role of visual processing speed for reading performance seems to go beyond DD pathology: first, in a recent TVA-based study Lobier et al. (2013) showed that, in typically developing children, the individual speed of visual processing predicted that of text reading. Thus, visual processing speed seems to have a central functional role in both pathological and normal reading development. Second, a visual processing speed reduction was documented also in acquired reading disorders in brain-damaged patients with simultanagnosia (Duncan et al., 2003). Therefore, also when reading development is completed, a severe slowness of processing speed might reduce the rate of information uptake below the limit required for normal reading performance. How do reductions in the TVA parameter processing speed relate to the reading difficulties in DD? Two well-established findings are compatible with the notion of reduced processing speed. One, the so-called “double deficit hypothesis” (Bowers and Wolf, 1993) is related to the results of Lobier et al. (2013). It describes a reduction in naming speed (for verbal or non-verbal material), in combination with a phonological deficit in DD. The second demonstrates a reading speed deficit in DD which is possibly based on slow decoding mechanisms, especially in regular orthographies (for discussion of underlying impairments, see Wimmer, 1993). Compared to processing speed, findings on VSTM storage capacity are, at first glance, less consistent in the studies reviewed here. In the adult-group study, VSTM storage capacity was comparable between the group with DD and controls (Stenneken et al., 2011). In contrast, a marked reduction in VSTM was revealed in the group of children with DD (Bogon et al., 2014) and in one of the children with DD studied by Dubois et al. (2010). Obviously, low VSTM storage capacity does not present a shared deficit in all persons with DD. At second glance, these inconsistencies might reflect an influence of academic achievement. The TVA-based group studies on children and on adults with DD differed concerning the academic levels of the participants. While the adults, despite persisting DD, had above-average academic achievement, the children were unselected with respect to their own or their parent’s academic achievements. Therefore, one could speculate that, in persons suffering from DD, a normal VSTM storage capacity might facilitate the compensation of DD-induced academic deficits while low VSTM storage capacity might induce a higher probability for academic failure. In support of this assumption, VSTM storage capacity in the group of children with DD was related to better reading performance (Bogon et al., 2014).

With regard to selectivity aspects of visual attention, adults with DD differed from controls in spatial weighting (Stenneken et al., 2011). The DD group did not show the typical pseudoneglect bias to the left (Bowers and Heilman, 1980; Jewell and McCourt, 2000) but rather a balanced distribution of weights. Interestingly, in children, such balanced weighting was present in both, DD and control group (Bogon et al., 2014). Thus, in normal participants, the TVA-based group studies (**Figure 2**), in line with findings from line bisection studies (Hausmann et al., 2003; Sireteanu et al., 2005), suggest that a leftward bias emerges or increases during development. In adults with DD, the absence of the spatial bias might be a primary deficit underlying DD. However, two lines of evidence suggest that it rather reflects a secondary consequence of reduced left-to-right reading experience in impaired readers. First, differences between adult left-to-right and right-to-left readers in line- and string-bisection performance imply that reading experience and cultural reading habits have an influence on pseudoneglect development (Chokron and Imbert, 1993; Chokron and de Agostini, 1995; Zivotofsky, 2004; Kazandjian et al., 2010). Second, the fact that a group of children showed the typical DD symptoms in the absence of spatial weighting differences from a control group (Bogon et al., 2014) quite obviously indicates that the onset of DD precedes that of spatial attention changes.

Concerning the second selectivity aspect assessed, the reviewed studies reviewed above indicate that top–down control is not impaired in DD (Stenneken et al., 2011; Bogon et al., 2014; see **Figure 2**). Thus, the TVA-based analyses do not support the previously suggested assumption that multi-element processing deficits in DD result from an inability to prioritize relevant over irrelevant information (Sperling et al., 2005; Roach and Hogben, 2007; Moores et al., 2011; Stevens et al., 2013).

## CONCLUSION

We summarized the results of studies that used TVA-based assessment of visual attentional parameters to examine a potential relevance of deficient visual attentional processing in DD. Taken together, in children and adults with DD (Dubois et al., 2010; Stenneken et al., 2011; Bogon et al., 2014) marked reduction in visual processing speed seem to be a core deficit in DD. Furthermore, less consistently documented reductions in VSTM storage capacity may have a modulating effect on word processing performance and written language acquisition. In addition, reduced left-to-right reading skills and training in persons with DD might impair the development of a slight leftward attentional bias that is typically observed in unimpaired adult readers. It is unknown whether this absence of pseudoneglect contributes to the persistent reading deficits in adulthood or whether it is an epiphenomenon without functional significance. In sum, findings from all rather recent parameter-based studies of DD point to significant reductions in general information processing efficiency as underlying mechanisms for impaired multi-element processing in DD. Moreover, recent studies of visual attentional span tasks support a visual—rather than an exclusively verbal or phonological—nature of the underlying deficit (Lobier et al., 2012; Valdois et al., 2012). Thus, parameter-based assessment offers new directions in investigating impaired visual attentional functions that seem to constitute an additional or even isolated deficit of DD, as previously suggested in subgroup-accounts of reading disorders (Morris et al., 1998; Heim et al., 2008; Menghini et al., 2010).

## Conflict of Interest Statement

The authors declare that the research was conducted in the absence of any commercial or financial relationships that could be construed as a potential conflict of interest.
